# 

**Published:** 1899-12

**Authors:** 


### SELECTIONS.

Article I.—What Patients Should Know. Dr. A. T. Bigelow..... 337
" II.—Some Mysterious Cases. D. W. Barker, M. D. S ..... 346
" III.—The Niagara Meeting..... 348
" IV.—A Plea for the Preservation of the Natural Teeth. C. N. Johnson, L. D. S., D. D. S ..... 358
" V.—Three Vital Subjects. Lin. C. Shecut, D. D. S..... 368
" VI.—The Wisconsin Decision..... 372
" VII.—Painless Dentistry..... 376

### COMMUNICATED.

International Dental Congress..... 378

### MONTHLY SUMMARY.

Under One Flag..... 381

Treatment of Abscess Swelling..... 382

Oil for Appendicitis..... 383

Extraction of Teeth ..... 383

To Remove Gutta Percha Points from Root-Canals..... 383

A School Inspection..... 384

A Counter-Irritant..... 384

To Give a Fine Finish to Gold..... 384

